# Scanning laser optical tomography for *in toto* imaging of the murine cochlea

**DOI:** 10.1371/journal.pone.0175431

**Published:** 2017-04-07

**Authors:** Lena Nolte, Nadine Tinne, Jennifer Schulze, Dag Heinemann, Georgios C. Antonopoulos, Heiko Meyer, Hans Gerd Nothwang, Thomas Lenarz, Alexander Heisterkamp, Athanasia Warnecke, Tammo Ripken

**Affiliations:** 1 Industrial and Biomedical Optics Department, Laser Zentrum Hannover e.V., Hannover Germany; 2 Cluster of Excellence “Hearing4all”, Hannover and Oldenburg, Germany; 3 Department of Otorhinolaryngology, Head and Neck Surgery, Hannover Medical School, Hannover, Germany; 4 Biofabrication for NIFE, Hannover, Germany; 5 Neurogenetics, Carl von Ossietzky University Oldenburg, Oldenburg, Germany; 6 Institute of Quantum Optics, Leibniz University of Hanover, Hannover, Germany; Aix Marseille University, FRANCE

## Abstract

The mammalian cochlea is a complex macroscopic structure due to its helical shape and the microscopic arrangements of the individual layers of cells. To improve the outcomes of hearing restoration in deaf patients, it is important to understand the anatomic structure and composition of the cochlea *ex vivo*. Hitherto, only one histological technique based on confocal laser scanning microscopy and optical clearing has been developed for *in toto* optical imaging of the murine cochlea. However, with a growing size of the specimen, e.g., human cochlea, this technique reaches its limitations. Here, we demonstrate scanning laser optical tomography (SLOT) as a valuable imaging technique to visualize the murine cochlea *in toto* without any physical slicing. This technique can also be applied in larger specimens up to cm^3^ such as the human cochlea. Furthermore, immunolabeling allows visualization of inner hair cells (otoferlin) or spiral ganglion cells (neurofilament) within the whole cochlea. After image reconstruction, the 3D dataset was used for digital segmentation of the labeled region. As a result, quantitative analysis of position, length and curvature of the labeled region was possible. This is of high interest in order to understand the interaction of cochlear implants (CI) and cells in more detail.

## Introduction

The anatomic structure of the cochlea is of high interest for efforts to improve the outcomes of hearing restoration. The *ex vivo* visualization of the mammalian cochlea can be achieved by several imaging techniques. A widespread technique for *ex vivo* imaging is serial histological sectioning. For that, the sample is immobilized by embedding in paraffin or by freezing for cryosections and then cut in sections with a constant thickness [1]. These sections are mounted on microscope slides and can be visualized using 2D imaging. To obtain sufficient contrast, additional stains are applied to the sections, such as hematoxylin and eosin [2]. 3-dimensional registration of these images can be performed to generate a volumetric dataset of the cochlea [3]. This method has been used to visualize the basilar membrane inside rodent cochlea [4,5]. The inner structures of the human cochlea such as the organ of Corti and the spiral ganglion cells have been analyzed as well [6,7]. Using transmission electron microscopy in combination with serial sectioning, the outer hair cells of the mammalian cochlea, which are responsible for the amplification of low-level sounds, have been visualized in 3D with a resolution down to some nanometers [8]. However, the samples can be damaged by physical slicing. Thus, cutting artifacts may occur and volumetric information can get lost. Especially in case of the cochlea with its helical structure, the slicing into single planes is not sufficient for 3D visualization and tracking. Other methods to visualize the cochlea in 3D are micro-computed tomography (μCT) and magnetic resonance imaging (MRI): μCT uses the absorption of X-rays inside the sample to generate a volumetric image. This method has been used in several studies to generate a model of the bony structure of the cochlea [9–11]. By using osmium tetroxide as an additional contrast agent, the soft tissue inside the cochlea has been visualized and used for modeling [12]. MRI has also been used as one further non-destructive method to visualize a sample in 3D [13,14]. However, CT and MRI do not allow a cell type specific labeling of the cochlea.

In contrast, fluorescence microscopy enables the use of antibody labeling, which tags a specific cell type with a certain fluorophore. Hardie *et al*. showed the specific labeling of the inner hair cells, which convert the sound wave information in an electrical signal, and visualization in the native sectioned cochlea by confocal microscopy [15]. One intrinsic limitation of optical imaging is its penetration depth in tissue. MacDonald *et al*. showed specific labeling of inner hair cells and spiral ganglion cells in an optically cleared mouse cochlea [16]. Clearing enables a larger penetration depth into the sample [17,18]. However, the working distance of an objective still represents a general limitation. An enlarged field of view can be achieved by orthogonal plane fluorescence microscopy. Several studies visualized a mouse and guinea pig cochlea by thin-sheet laser imaging microscopy and a nonspecific fluorescence inside the sample [18–21].

A promising method for cochlea imaging is scanning laser optical tomography (SLOT) [22]. This tomography-based technique enables the visualization of transparent samples up to several centimeters as shown for the murine lung [23]. Furthermore, SLOT benefits from more efficient signal collection compared to other optical tomography technologies [24], which is particularly valuable at low signal levels. In this study, we show the capability of SLOT to image specifically labeled regions inside the murine cochlea *in toto*, without the need for physical slicing of the sample. After segmentation, quantitative information can also be extracted from the datasets. Moreover, multichannel fluorescence images can be generated to visualize hair cells and spiral ganglion cells in the same sample.

## Material and methods

### Animals

In total, three murine cochleae of NMRI wild type mice (postnatal day 27) were used for this study. After CO_2_ anesthesia, mice were decapitated in accordance with the German animal welfare act and the cochleae were isolated. After removal of the stapes, a small hole was made in the apex of the cochlea. A small syringe was used for slow perfusion with 4% paraformaldehyde through the round window and the hole in the apex. The fixation needed an incubation time of 15 minutes on ice. Afterwards, each cochlea was washed three times with PBS. All experiments were carried out in accordance with the European communities Council Directive (86/609/ECC), the German Animal Protection law and approved by the local animal care and use committee (LAVES, Oldenburg). Protocols also followed the NIH guide for the care and use of laboratory animals.

### Decalcification and antibody staining

**Fig 1:**
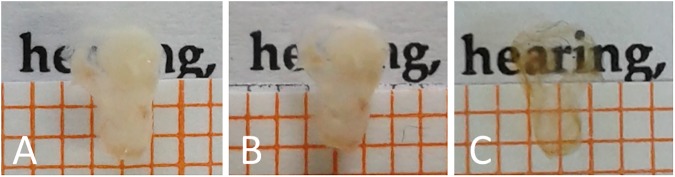
The same explanted murine cochlea (sample 1) is shown during different steps of sample preparation. (A) Cochlea after fixation. (B) Cochlea after decalcification. (C) Cochlea after clearing. The spacing of the grid amounts to 1 mm.

For decalcification, cochleae were stored in a 10% ethylenediaminetetraacetic acid (EDTA, Sigma Aldrich, USA) phosphate buffered saline (PBS) solution with pH 7.4 for four days. This slowly removed the calcium from the sample by chelation (compare Fig 1A and 1B). To remove EDTA, samples were washed three times for 10 minutes in washing buffer (WB, 0.1% Triton-X (Sigma Aldrich, Germany) in PBS). Subsequently, samples were transferred into image-iT® FX signal enhancer (Thermo Fisher Scientific, Germany) for 30 minutes to reduce unwanted background signal. A fourth washing step in WB followed, ensued by the transfer of the samples into a blocking solution (BS, 1% Triton-X, 10% fetal calf serum (PAN Biotech GmbH, Germany) and 0.5% bovine serum albumin (Sigma Aldrich, Germany) in PBS). The samples remained in this solution for four hours and were subsequently incubated in BS containing primary antibodies if designated (see Table 1). After incubation for three days, three steps of washing in WB and subsequent incubation in BS plus secondary antibodies (see Table 1) for another three days followed. To remove unbound antibodies, three further washing steps in WB were conducted. All incubation steps were performed at room temperature and at gentle agitation.

**Table 1:** Used primary and secondary antibodies are listed with dilution ratio for the individual samples.

	Primary antibody	Secondary antibody
Sample 1	Anti-200 kDa neurofilament chicken, 1:10000 (Abcam plc, UK)	Goat anti-chicken Cy3, 1:500 (Jackson Immuno Resaerch Laboratories Inc., USA)
Sample 2	-	Goat anti-chicken Cy3, 1:500(Jackson Immuno Resaerch Laboratories Inc., USA)
Sample 3	Anti-200 kDa neurofilament rabbit, 1:200(Sigma-Aldrich, USA)	Goat anti-rabbit Cy3, 1:500(Jackson ImmunoResaerch Laboratories Inc., USA)
	Anti-otoferlin mouse, 1:200(Abcam plc, UK)	Goat anti-mouse Cy5, 1:500(Santa Cruz Biotechnology Inc., USA)

### Clearing

For dehydration, stained samples were stored overnight in 70% ethanol followed by 30 minutes incubation in 95% ethanol and two further steps in 100% ethanol for at least two hours. Clearing was achieved by MSBB (mixture of methyl salicylate and benzyl benzoate, also known as Spalteholz fluid [17,18]) with refractive index of n = 1.552, starting with incubation of the samples in a solution of 50% ethanol and 50% MSBB for four hours and three subsequent steps of incubation in 100% MSBB overnight, for four hours and for two hours (compare Fig 1C). All steps were performed at room temperature and gentle agitation.

### SLOT imaging

The principle of SLOT has been described in detail elsewhere [24]. Briefly, the beams of a 520 nm and a 635 nm laser diode are coupled into a single mode fiber (P3-460, Thorlabs Inc, USA) to generate a homogeneous TEM 00 beam profile. Afterwards, the laser light is directed through a zoom telescope (H10Z1218MP, computar, CBC AMERICAS Corp., USA) to adjust the numerical aperture of the illumination system and directed onto an x-y-scanning mirror system (ProSeries II Scan Head, Cambridge Technology, USA). This system is positioned in the back focal plane of a telecentric F-Theta lens (S4LFT0080/121, Sill Optics GmbH & Co. KG, Germany) to achieve a parallel displacement of the laser beam. The sample was positioned in the focal plane of the scanning lens and connected to a stepping motor (M-060.PD, Physik Instrumente GmbH, Germany) which allowed for a 360° rotation of the sample inside a cuvette. This cuvette was filled with a mixture of MSBB matching the refractive index of the sample. Extinction of the scanning laser beam was measured by a photo diode (PDA100A, Thorlabs Inc, USA) behind the cuvette. The fluorescence signal was collected orthogonally to the illumination axis and detected by a photomultiplier tube (R3896, Hamamatsu Photonics K.K., Japan) with the corresponding bandpass filter (F37-565 for Cy3 and F37-679 for Cy5, AHF, Germany). Fluorescence measurements of different fluorophores were performed successively. Projection images with 2000 pixels in x-direction and 1800 pixels in y-direction (1.66 μm/pixel) were generated for every 0.3° rotational step of the sample. The acquisition time per image amounts to 7 s, which results in a total acquisition time of about 140 min. The theoretical optical spatial resolution of the system amounts to 5.6 μm. The set of projection images was subsequently reconstructed to a 3D tomographic dataset using a filtered back projection algorithm from the open source software IMOD [25]. After data acquisition and reconstruction, individual digital slicing was performed on the 3D dataset. All samples were imaged using these parameters.

### Image processing

A maximum intensity projection (MIP) was applied in Fiji [26,27] to each reconstructed 3D dataset to visualize connected structures that appear in multiple layers. The scanned area shifts and scales slightly due to chromatic aberrations depending on the excitation wavelength. To enable correct overlay of the two excitation channels, the general registration algorithm (BRAINS) from the open source software 3D Slicer was used [28]. Since both fluorophores label different areas inside the sample, the extinction of both channels was used to perform registration. The resulting image transformation is then exported and applied to the corresponding fluorescence image. Segmentation of the neurofilament was performed by a semi-automated algorithm using the open source software ITK-SNAP [29]. The length of the segmented area was measured by the Fiji plugins Skeletonize3D and AnalyzeSkeleton [30]. To measure the curvature of the segmented area, the segmentation was sampled semi-automatically in Fiji. Curvature measurements were performed in Octave using the circlefit3d program [31,32].

## Results and discussion

**Fig 2:**
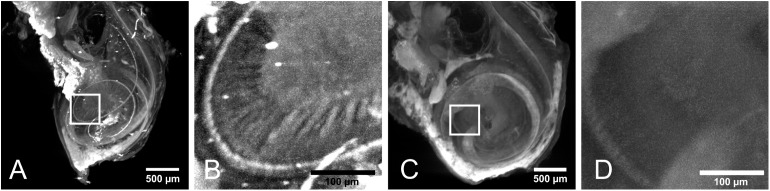
Maximum intensity projections (MIP) were performed on the reconstructed data of samples 1 and 2. (A) MIP of sample 1. The labeled neurofilament appears as a helical shape inside the cochlea. (B) Higher magnification of the highlighted area in A. The dendrites of the spiral ganglions are visible. (C) Negative control (sample 2). Only autofluorescence and nonspecific binding shows the outer shape of the cochlea. (D) Higher magnification of the highlighted area in C.

Fig 2 shows the maximum intensity projections (MIP) of the cochlea with labeled neurofilament and its negative control (samples 1 and 2 in Table 1) excited at 520 nm. In the labeled sample 1, the spiral structure is visible in the MIP (see Fig 2A). This is due to the dense accumulation of neurons in the Rosenthal’s canal connecting to the inner and outer hair cells within the organ of Corti. By enlarging part of the image, the dendrites of the spiral ganglion inside the cochlea are visible (see Fig 2B). None of these structures are visible in the negative control without specific labeling for neurofilament (see Fig 2C and 2D). Besides the specific labeling, strong autofluorescence of the sample appears in this channel, which is most likely due to protein crosslinking after fixation. This, however, does not disturb imaging of the spiral ganglion cells. Also, the general morphological shape of the cochlea is visible without the use of any label in sample 2 (negative control). The bright spots in both samples are most likely due to agglomerations of dye or strongly scattering air bubbles inside the sample.

**Fig 3:**
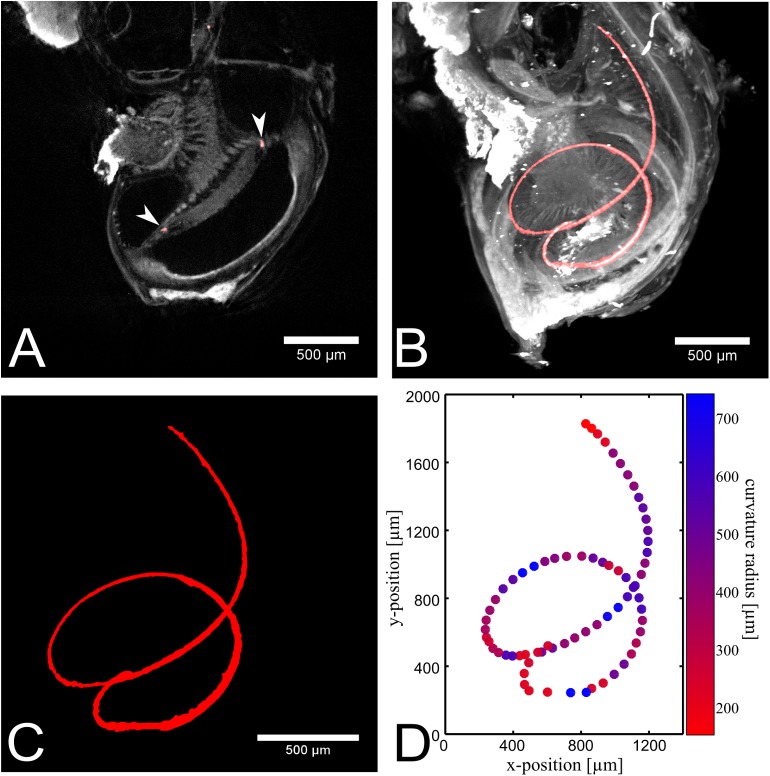
Segmentation of the neurofilament labeling in sample 1. (A) One slice of the segmentation is overlaid with the reconstructed data. Due to the 3D helical shape, the neurofilament appears in a single slice just as three single points (see arrow heads). (B) Maximum intensity projection of the segmentation, overlayed with the dataset. (C) The segmented area is visualized individually. (D) The extracted points of the segmented area are plotted. The curvature of the neurofilament is indicated by the color map. Blue shows a large curvature radius and red a small curvature radius in μm.

Fig 3 shows the segmentation of the dense accumulation of neurons in the organ of Corti of sample 1. Fig 3A shows a cross section of the cochlea in grayscale and the segmented area in red (indicated by white arrow heads). Here, the helical shape appears as 3 single spots in contrast to the maximum intensity projection (compare Fig 3B and 3C). The segmentation enables the measurement of the neurofilament length, which was6.64 mm for this murine cochlea. This is in good agreement with a previously published value of 6.8 mm [33]. Furthermore the curvature radii were determined for multiple parts of the segmentation, which are displayed in a color coding in Fig 3D. For curvature measurements all three spatial dimensions are considered. Since a 2 dimensional projection is shown here, strong curvatures into the image plane are not obvious. The ratio of the curvature radii at the base and the apex was150 μm/260 μm = 0.58. In contrast to other studies ([33]), we measured a smaller curvature radius at the base compared to the apex. This mismatch is due to the fact that we measured the curvature in three dimensions, and not only a projection in two dimensions as done previously [33]. Measuring the curvature radii in a 2D MIP resulted in a radii ratio of 1819 μm/693 μm = 2.6. This is in better agreement to other studies ([33]) and shows the sensitivity of measured curvatures to the projection angle. Furthermore, we were able to measure the curvature radius at multiple positions of the sample in contrast to the two point measurement at base and apex.

**Fig 4:**
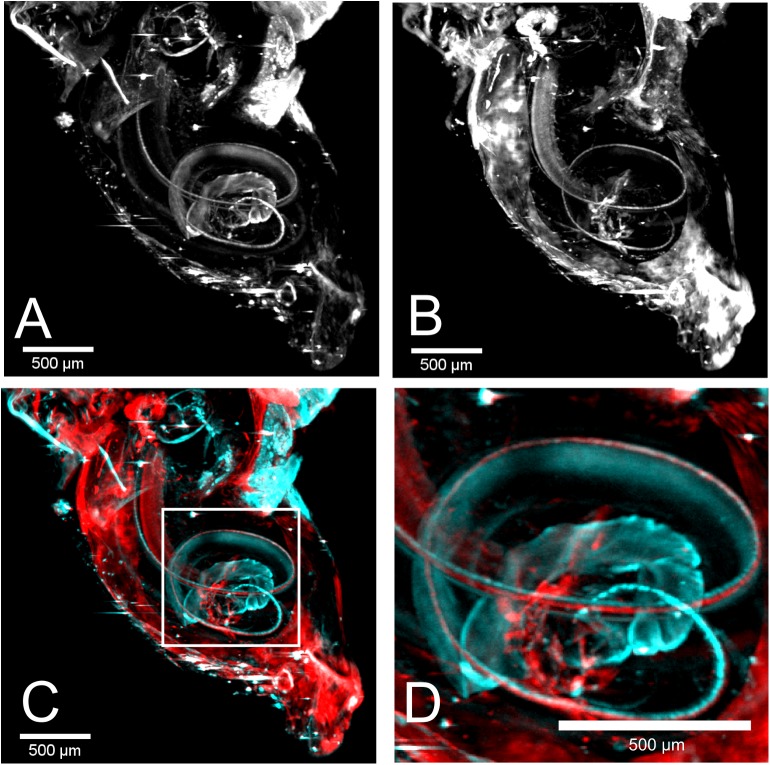
Double labeling of cochlea 3 with a combination of two antibodies. (A) The MIP of the neurofilament excited at 520 nm is shown. The helical shape is visible as for sample 1. (B) The labeled inner hair cells also appear in a helical shape in the MIP excited at 635 nm. (C) An overlay of neurofilament (cyan) and hair cells (red) is shown. (D) Higher magnification of the highlighted region in C. Due to the overlay the arrangement of neurofilament and hair cells becomes visible.

The measurements of the individual fluorescence channels were performed consecutively. Fig 4A and 4B show a MIP of the reconstructed data visualizing the labeled neurofilament and inner hair cells respectively from sample 3. Both appear in a helical arrangement due to the architecture of the cochlea. Similar to Fig 2, strong autofluorescence is visible in Fig 4A, since both measurements were performed using the 520 nm laser source. The even stronger background signal in Fig 4B, which was generated by the 635 nm laser, was most probably due to nonspecific binding of the secondary antibody since no autofluorescence was detected at this excitation wavelength in untreated samples. Due to this strong background fluorescence, high intensities appear on the surface of the cochlea. Therefore, the MIP in Fig 4 was applied to the inner part of the cochlea only to remove overlaying signal from the surface. The registration was performed as explained above. The corrected overlay of both channels is shown in Fig 4C. By enlarging the helical part, the side-by-side arrangement of neurofilament and inner hair cells becomes visible (see Fig 4D).

SLOT in combination with immunolabeling provides three major advantages for imaging cochleae: First, it allows 3D *in toto* visualization. Most other optical imaging techniques, such as confocal microscopy, provide a higher resolution but suffer from a small field of view and require cutting of the sample. Cutting might be accompanied by distortion and changes of the slices themselves. This can be excluded by *in toto* imaging using SLOT. Second, due to the isotropic resolution of SLOT, isotropic 3D segmentation is possible. This is the base for quantitative geometrical analysis of the labeled region. Third, due to the rotational scanning, SLOT can be combined with partially non-transparent samples [34], which is harder to realize using methods such as confocal microscopy or light sheet based techniques due to shading effects. These three points in combination enable the visualization of the cochlear implant (CI) inside the inner ear. This way, the geometry and shape of the implant itself in respect to biological structures can be analyzed. If high resolution images are necessary, like for distinguishing individually the labelled sensory cells, other techniques as multiphoton microscopy or electron microscopy need to be consulted.

## Conclusion

In this paper, we show that SLOT is well suited to visualize the cochlea in three dimensions without any physical slicing. After reconstruction, every plane in any orientation can be analyzed individually. SLOT cannot be applied to not-cleared cochlear samples, as it is the case for μCT, but therefore it provides the ability to show a more detailed contrast for tissue components. Furthermore, due to the nature of immunolabeling, multiple targets can be labeled in the same sample, which allows co-localization analysis. This enables the investigation of animal models with neuronal defects in respect to neuronal modifications. In addition, segmentation can be used and help to visualize and quantify areas of interest inside the cochlea. Since SLOT can also be applied to partially non-transparent samples, the position of an inserted CI will provide valuable information for implant design. Additional labeling of the cochlea with cell-specific antibodies would enable the segmentation of neurons and implant in order to measure the distance between both. Thus, molecular changes due to a specific implant design can be understood in more detail. Cochleae inserted with different types of implants could be imaged using SLOT and the presence or absence of cell type specific signals can indicate the cell preservation for the respective hearing restoration strategy. These results may aid for the improvement of the hearing quality of CI users.

## Supporting information

S1 File Coordinates of the extracted points of the segmentation and corresponding curvature radii. (TXT)
